# Twenty-eight divergent polysaccharide loci specifying within- and amongst-strain capsule diversity in three strains of *Bacteroides fragilis*

**DOI:** 10.1099/mic.0.042978-0

**Published:** 2010-11

**Authors:** Sheila Patrick, Garry W. Blakely, Simon Houston, Jane Moore, Valerie R. Abratt, Marcelo Bertalan, Ana M. Cerdeño-Tárraga, Michael A. Quail, Nicola Corton, Craig Corton, Alexandra Bignell, Andrew Barron, Louise Clark, Stephen D. Bentley, Julian Parkhill

**Affiliations:** 1 Centre for Infection and Immunity, School of Medicine, Dentistry and Biomedical Sciences, Queen's University Belfast, Medical Biology Centre, 97 Lisburn Road, Belfast BT9 7BL, UK; 2 Institute of Cell Biology, University of Edinburgh, Darwin Building, Kings Buildings, Edinburgh EH9 3JR, UK; 3 Department of Molecular and Cell Biology, University of Cape Town, South Africa; 4 The Pathogen Sequencing Unit, The Wellcome Trust Sanger Institute, Hinxton, Cambridge CB10 1SA, UK

## Abstract

Comparison of the complete genome sequence of *Bacteroides fragilis* 638R, originally isolated in the USA, was made with two previously sequenced strains isolated in the UK (NCTC 9343) and Japan (YCH46). The presence of 10 loci containing genes associated with polysaccharide (PS) biosynthesis, each including a putative Wzx flippase and Wzy polymerase, was confirmed in all three strains, despite a lack of cross-reactivity between NCTC 9343 and 638R surface PS-specific antibodies by immunolabelling and microscopy. Genomic comparisons revealed an exceptional level of PS biosynthesis locus diversity. Of the 10 divergent PS-associated loci apparent in each strain, none is similar between NCTC 9343 and 638R. YCH46 shares one locus with NCTC 9343, confirmed by mAb labelling, and a second different locus with 638R, making a total of 28 divergent PS biosynthesis loci amongst the three strains. The lack of expression of the phase-variable large capsule (LC) in strain 638R, observed in NCTC 9343, is likely to be due to a point mutation that generates a stop codon within a putative initiating glycosyltransferase, necessary for the expression of the LC in NCTC 9343. Other major sequence differences were observed to arise from different numbers and variety of inserted extra-chromosomal elements, in particular prophages. Extensive horizontal gene transfer has occurred within these strains, despite the presence of a significant number of divergent DNA restriction and modification systems that act to prevent acquisition of foreign DNA. The level of amongst-strain diversity in PS biosynthesis loci is unprecedented.

## INTRODUCTION

The obligately anaerobic Gram-negative bacterium *Bacteroides fragilis* is an important member of the open-ended culture system of the normal resident human gastrointestinal (GI) faecal microbiota, in which the genera *Bacteroides* and related *Parabacteroides* predominate both by culture (Patrick *et al.*, 2009) and metagenomic sequencing (Qin *et al.*, 2010). Since the late 1800s, *B. fragilis* (previously *Bacillus fragilis*) has also been recognized as an important opportunistic pathogen. It is the major obligately anaerobic Gram-negative bacterium isolated from abscess and soft-tissue infections that arise from contamination by the GI microbiota of normally uncolonized body sites; *B. fragilis* is estimated to account for only between 4 and 13 % of the normal human faecal *Bacteroides* microbiota by culture and is less abundant than other members of the Bacteroidetes, as demonstrated by metagenomic analysis, but is present in 63–80 % of *Bacteroides* infections. These include peritonitis subsequent to rupture of an inflamed appendix, serious gynaecological sepsis and brain abscesses (Patrick, 2002; Patrick & Duerden, 2006). *B. fragilis* is also the most common cause of anaerobic bacteraemia, with a potential mortality of up to ∼30 % (Cheng *et al.*, 2009). Prior to the antibiotic era, such infections severely limited the success of GI and gynaecological surgery due to the high rates of patient death (Patrick & Duerden, 2006). Metronidazole is generally the most effective antibiotic for both treatment and prophylaxis; a recent worrying development, however, is the report of potentially lethal multi-drug-resistant strains (Katsandri *et al.*, 2006; Wareham *et al.*, 2005). In contrast to *B. fragilis*, other Bacteroidetes more abundant in the GI tract, such as *Bacteroides vulgatus* and *Parabacteroides distasonis* (formerly *Bacteroides distasonis*; Sakamoto & Benno, 2006), are rarely isolated from infection (Patrick & Duerden, 2006). Characteristics of *B. fragilis* that may play a role in its success as an opportunistic pathogen include the release of degradative enzymes such as fibrinogenolysin (Houston *et al.*, 2010), enterotoxin production, evasion of complement-mediated killing and phagocytosis, the induction of abscess formation, and extensive within-strain variation of surface proteins and polysaccharides (PSs) (Wexler, 2007). Multiple invertible promoters play a key role in generating the observed antigenic variation (Cerdeño-Tárraga *et al.*, 2005; Patrick *et al.*, 2003). The marginal electron-dense layer, or micro-capsule (MC), of approximately 35 nm in size, outwith the outer membrane and not visible by light microscopy (Patrick *et al.*, 1986; Lutton *et al.*, 1991), is linked to complement resistance, and at least one of these antigenically variable PSs is associated with abscess formation (Wexler, 2007). In addition to MC, antigenically distinct and within strain-variable large capsules (LCs) and small capsules (SCs) are visible by light microscopy when cultures are grown in a glucose-defined medium (Patrick *et al.*, 1986). The LC is associated with resistance to phagocytic uptake and killing (Reid & Patrick, 1984). A putative PS chain length-determining Wzz protein homologue is involved in the production of high-molecular-mass polysaccharides (HMMPS) associated with the MC; a *wzz* deletion mutant in NCTC 9343 does not produce HMMPS, but still produces SC and LC (Patrick *et al.*, 2009). Phase variation of LC production is also under invertible promoter control (Patrick *et al.*, 2009).

We now present the complete genome sequence of a *B. fragilis* plasmid-free spontaneous rifampicin-resistant mutant, 638R (Privitera *et al.*, 1979; also known as TM400; Tally *et al.*, 1982), which was generated at the Institut Pasteur (Paris, France) from a clinical isolate originating from Chicago, USA (Stiffler *et al.*, 1974). Comparison of 638R with two published *B. fragilis* genome sequences, NCTC 9343 (Cerdeño-Tárraga *et al.*, 2005), isolated from peritoneal infection in London (UK), and a blood culture isolate, YCH46, isolated in Yamaguchi (Japan; Kuwahara *et al.*, 2004), reveals an unprecedented diversity of PSs amongst the three strains.

## METHODS

### Bacterial strains and culture conditions.

*B. fragilis* 638R (isolated from an abdominal abscess), kindly gifted by C. J. Smith, East Carolina University, USA, was derived from a culture supplied to him by M. Sebald (Institut Pasteur, Paris, France) in 1983. *B. fragilis* YCH46, a bacteraemia isolate from Yamaguchi Prefecture (Japan), was kindly gifted by T. Kuwahara, University of Tokushima (Japan). An early freeze-dried stock culture of *B. fragilis* National Collection of Type Cultures (NCTC) 9343, originally isolated from an abdominal infection at St Bartholomew's Hospital, London, UK, in 1955, was obtained from the NCTC, London, UK.

*B. fragilis* was cultured in either supplemented brain heart infusion (BHI-S) or glucose defined medium (DM; Van Tassell & Wilkins, 1978). *B. fragilis* was cultured in a MACS MG-1000 anaerobic workstation (Don Whitley) at 37 °C in an atmosphere of 80 % nitrogen, 10 % carbon dioxide and 10 % hydrogen. Clinical isolates (*n*=23) and GI tract isolates (*n*=9) were cultured in DM broth, and the proportion of capsulate cells was estimated by five random field counts (Patrick & Reid, 1983). Enrichment of populations with different sizes of capsules was carried out using discontinuous Percoll density-gradient centrifugation, as previously described (Patrick & Reid, 1983; Patrick *et al.*, 2009).

### *B. fragilis* 638R complete genome sequencing.

*B. fragilis* 638R was grown in DM as described above, and DNA was isolated using a modification of the basic protocol for preparation of genomic DNA from bacteria described by Ausubel *et al.* (1992). In brief, bacterial cells were lysed in 10 mM Tris/HCl/1 mM EDTA buffer (pH 8.0) containing SDS (0.5 %), lysozyme (4 mg ml^−1^) and proteinase K (0.1 mg ml^−1^). PS was precipitated using cetyltrimethylammonium bromide (CTAB) and DNA was extracted with chloroform : isoamyl alcohol and phenol : choloroform : isoamyl alcohol mixtures. DNA was precipitated using 2-propanol, spooled out with a glass rod and washed in ethanol. The initial genome assembly was obtained from 86 560 paired-end sequences (giving ninefold coverage) derived from two pUC18 genomic shotgun libraries (with insert sizes ranging from 1.4 to 3.6 kb) using BigDye terminator chemistry on ABI 3700 automated sequencers. A total of 1474 paired-end sequences from a pBACe3.6 library were used as a scaffold. All identified repeats were bridged by read-pairs or end-sequenced PCR products. A further 14 870 sequencing reads were generated during finishing. The sequences were assembled, finished and annotated as described previously, using Artemis to collate data and facilitate annotation (Cerdeño-Tárraga *et al.*, 2005). The DNA and encoded protein sequences of related species were compared using the Artemis Comparison Tool (ACT; Carver *et al.*, 2005). A percentage identity cut-off was not set for the ACT comparisons. Whole-genome comparisons were performed using blast with default parameters, and all matches with a blast score >100 were collected and displayed. Non-homologous regions showed no matches above this threshold. Orthologous gene sets were calculated by reciprocal best match fasta comparisons, with subsequent manual curation. Pseudogenes had one or more mutations that would ablate expression; each of the inactivating mutations was subsequently checked against the original sequencing data.

Regions associated with PS biosynthesis were numbered in the primary annotation of the NCTC 9343 genome sequence; these numbers have been retained along with the letters subsequently designated for PS loci A–J (Patrick *et al.*, 2003; Cerdeño-Tárraga *et al.*, 2005) to enable cross-reference. Additional loci reported herein have been assigned both letter and number designations.

### Microscopy.

Bacteria grown in DM broth to late-exponential phase were fixed with glutaraldehyde and osmium tetroxide, and prepared for transmission electron microscopy as previously described (Patrick *et al.*, 1986). Capsule smears were performed on DM-grown cultures using Eosin–carbol fuchsin negative stain. Hybridoma cell lines secreting mouse mAb were generated by inoculation of whole bacterial cells, as previously described (Lutton *et al.*, 1991) under UK Government Home Office Personal and Project Licences and with local ethical approval. Immunofluorescence labelling was carried out as described by Lutton *et al.* (1991) with the following modifications. Bacterial cells, grown in DM broth, were suspended at OD_600_ 0.3 (∼5×10^8^cells ml^−1^) in PBS. Bacterial suspension (10 μl) was applied to a Teflon-coated multiwell slide (ICN Biomedicals). The slides were then dried at 37 °C, and cells were fixed by submerging in 100 % methanol at –20 °C for 20 min. After air drying, the cells were immunolabelled with mAb hybridoma supernatants (30 μl of a 1 : 10 dilution in PBS), incubated at 37 °C in a humidified container for 45 min, and then washed in PBS with agitation for 20 min. Secondary antibody, 1 : 100 goat anti-mouse IgG–FITC (Sigma), and 4 % (v/v) Evans blue counter stain in PBS (30 μl) were added to each well. Slides were incubated at 37 °C in a humidified container for 45 min and washed as before, mounted in glycerol–PBS anti-bleaching mounting fluid (Citifluor, Agar Scientific) and examined at ×1000 magnification (Leitz Ortholux fluorescence microscope). Images were captured using a Nikon DMX 1200 digital camera and Lucia G/F software.

### PCR amplification of the LC invertible promoter region.

Primers were designed which amplified the upstream region of BF2782 with the promoter in both the ON and OFF orientations, as follows: BF2782 ‘Promoter ON’ primers, BF2782 ON-For (5′-AAAAAAGGATCCATTAGGTAATAAATGCGGAATAGCG-3′) and BF2782 ON-Rev (5′-AAAAAAGCATGCAACTCTGTGTTCTCTGTGGTG-3′). This primer set amplifies a 1.85 kb DNA sequence upstream of BF2782 which has the promoter only in the ON position with respect to the BF2782 start codon; BF2782 ‘Promoter OFF’ primers, BF2782 OFF-For (5′-AAAAAAGGATCCCACTTAATAGAAATATTAGTC-3′) and BF2782 OFF-Rev (5′-AAAAAAGCATGCAACTCTGTGTTCCTCTGTGGTG-3′). This primer set amplifies a 1.75 kb DNA sequence upstream of BF2782 which has the promoter only in the OFF position with respect to the BF2782 start codon. DNA extraction and PCR were carried out as detailed in Patrick *et al.* (2009), with the following modifications. Purified genomic template DNA from each of the enriched capsular populations and strains 638R and YCH46 was diluted in autoclaved ultrapure water to give final concentrations of 100 μg ml^−1^. PCR mixtures were prepared using Platinum *Taq* High Fidelity DNA polymerase (Invitrogen) according to the manufacturer's instructions. The reaction mixtures were incubated at 94 °C for 3 min followed by 35 cycles of PCR amplification at 94 °C for 30 s, annealing at 60 °C for 15 s, extension at 68 °C for 1 min, and a final extension step at 68 °C for 10 min. PCR products were detected as described in Patrick *et al.* (2009).

### Transformation of strain 638R with pVA2198-BF2782.

Transformation was performed using pVA2198-BF2782, with the bacteria maintained in an anaerobic atmosphere throughout, as described in Patrick *et al.* (2009), with the following modifications. Under anaerobic conditions, *B. fragilis*, Ocr protein (8–16 μg), and plasmid (3.25 μg of pVA2198-BF2782 per electroporation) were added to the electroporation cuvette and mixed. As positive controls, *B. fragilis* 638R and NCTC 9343 were also electroporated with pVA2198 (500 ng plasmid and 8–16 μg Ocr protein). Transformed cells were initially plated onto BHI-S agar plates containing 10 μg erythromycin ml^−1^ with or without 20 μg rifampicin ml^−1^. Transformants were subcultured by patch plating individual colonies onto glucose DM agar plates containing 10 μg erythromycin ml^−1^ with or without 20 μg rifampicin ml^−1^ prior to performing capsule smears and microscopy.

## RESULTS AND DISCUSSION

### Within-strain antigenically variable MC comparisons

**Fig. 1.**
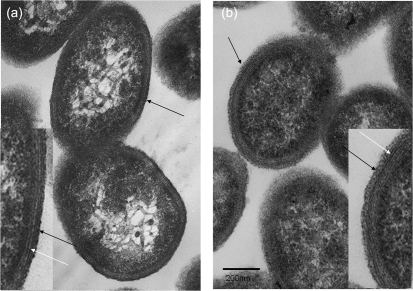
Electron micrographs of ultrathin sections of *B. fragilis*. (a) NCTC 9343 MC-enriched population; (b) *B. fragilis* 638R. Note the presence of an electron-dense layer (black arrow) outwith the outer membrane (white arrow) in both strains. Bar, 200 nm.

**Fig. 2.**
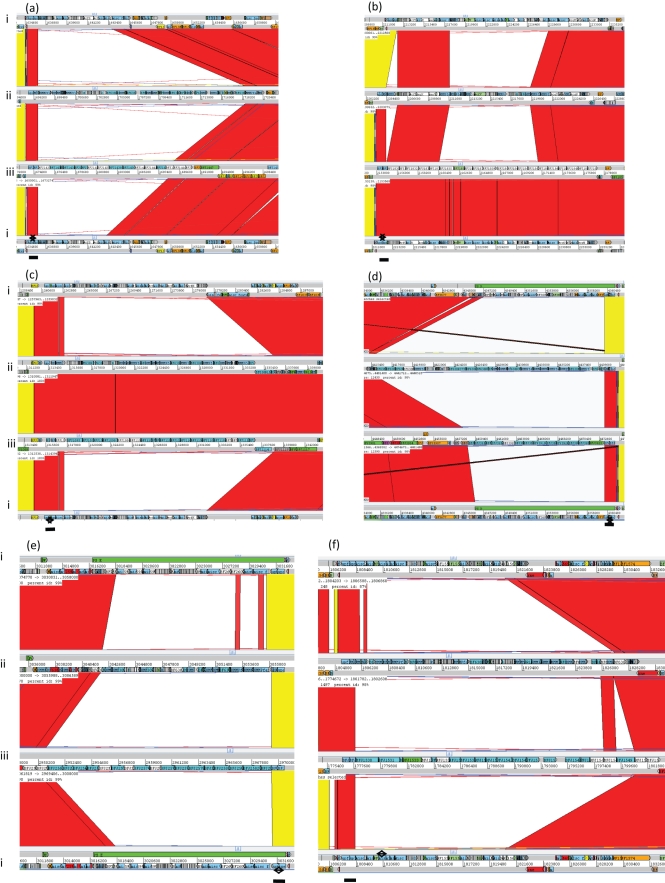

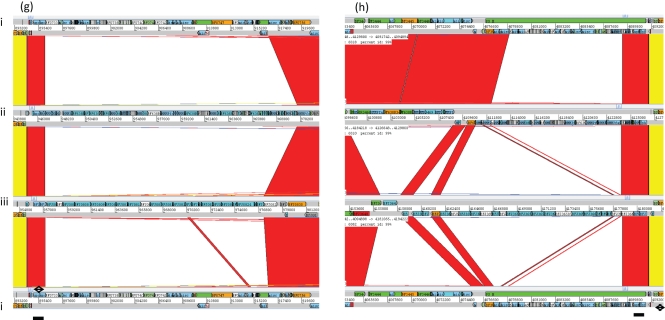
Four-way ACT genome comparison of within-strain variable MC-associated loci. (a) PSA/2, (b) PSB/4, (c) PSC/8, (d) PSD/5, (e) PSE/1, (f) PSF/6, (g) PSG/7, (h) PSH/3 of *B. fragilis.* (i) NCTC 9343, (ii) 638R, (iii)YCH46. Red or yellow coloration between sequences indicates sequence identity, no colour indicates divergent sequence, dark blue indicates an inverted promoter region. Line, conserved transcriptional regulator region. Note: sequence identity was only evident between strains NCTC 9343 and YCH46 at locus PSB/4, and strains 638R and YCH46 at locus PSC/8.

Within-strain antigenic variation of MC expression, driven by invertible promoters, is evident in *B. fragilis* NCTC 9343 and a range of other clinical isolates by reaction with a suite of mAbs. The rate of DNA inversion is sufficiently rapid to generate variants during growth of a single colony on glucose DM agar; thus, within a single population there are both promoter ON and OFF bacterial cells for multiple loci. As a result, immunofluorescence microscopy reveals, within a single population, labelling of variable numbers of bacterial cells with multiple different mAbs (Patrick *et al.*, 1999). *B. fragilis* 638R, however, does not react with the suite of mAbs that react with the MC of NCTC 9343 and also with a range of isolates from Northern Ireland (Patrick *et al.*, 1995); nevertheless, an electron-dense MC outwith the outer membrane is clearly evident in 638R by electron microscopy (Fig. 1). As previously described for NCTC 9343 (Patrick *et al.*, 2003; Cerdeño-Tárraga *et al.*, 2005), eight separate MC biosynthesis loci were also identified in the 638R genome, each characterized by the presence of putative O-antigen flippase genes (*wzx*) and polysaccharide polymerases (*wzy*), indicating the production of PSs, exported by Wzy-dependent methods (Whitfield, 2006). Whole-genome comparison, using ACT, revealed conservation of the upstream promoter regions, including the invertible regions associated with seven of the loci (Patrick *et al.*, 2003). As in NCTC 9343, there is evidence of active DNA inversion in the genome of the 638R population from which the DNA used for genome sequencing was extracted. Invertible promoters in both the ON and OFF positions are present in the 638R shotgun sequence data. In addition, the invertase *finA* (Patrick *et al.*, 2003) 638R 2788, equivalent to NCTC 9343 BF2779, which drives the DNA inversion at these loci, is conserved. The putative transcriptional regulators *up(a-h) Z* and *up(a-h) Y*, located at the start of seven of the PS biosynthesis loci, are also conserved, with the exception of the C-terminal region of the 638R *upbZ*. There is, however, a striking lack of DNA sequence identity between strain 638R and strain NCTC 9343 in the remainder of each of the loci as revealed by ACT analyses (Fig. 2a–h, Table 1). Within the non-identical regions, genes putatively involved in PS formation are present, potentially representing a different set of eight PSs in strain 638R. This divergence is likely to explain the lack of cross-reactivity of 638R with the NCTC 9343 antibodies. The PS-associated region PSJ/10, which encodes the only Wzz homologue, and which is involved in the generation of HMMPS associated with the MC, but not SC or LC formation (Patrick *et al.*, 2009), is however conserved amongst all three strains (results not shown).

**Fig. 3.**
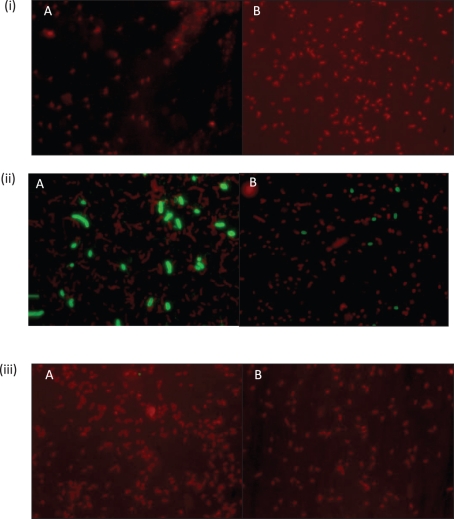
Immunofluorescence microscopy of *B. fragilis* strains. (i) NCTC 9343, (ii) 638R and (iii) YCH46 reacted with two representative mAbs, (A) and (B), raised against *B. fragilis* 638R, followed by anti-mouse FITC-conjugated secondary antibody, counter-stained with Evans blue. Exposure for fluorescein (green) image taken after shift of microscope slide (×100 objective). Note the within-strain variation in epitope expression in strain 638R and the lack of cross-reactivity with NCTC 9343 and YCH46.

**Fig. 4.**
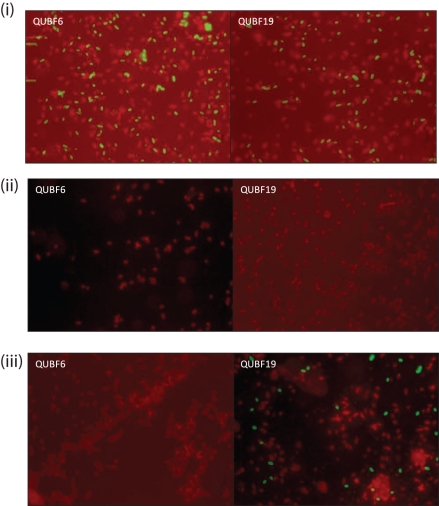
Immunofluorescence microscopy of *B. fragilis* strains. (i) NCTC 9343, (ii) 638R and (iii) YCH46 reacted with mAbs raised against NCTC 9343 followed by anti-mouse FITC-conjugated secondary antibody, counter-stained with Evans blue. Exposure for fluorescein (green) image taken after shift of microscope slide (×100 objective). QUBF6 reacts with PSE/1, and QUBF19 reacts with PSB/4.

Seven mAbs, generated using whole cells of 638R, reacted with HMMPS from 638R as demonstrated by proteinase K digestion, PAGE and immunoblotting (results not shown). Immunofluorescence microscopy revealed within-population antigenic variation in 638R, but not cross-reaction with NCTC 9343 (Fig. 3). Further genomic comparison with the previously sequenced Japanese strain, YCH46, which also contains eight MC loci, revealed only two shared loci amongst the three strains; 638R PSC/8 is shared with YCH46, and the NCTC 9343 PSB/4 locus is shared with YCH46. The existence of conserved loci between NCTC 9343 and YCH46, as observed using ACT, was confirmed experimentally by immunofluorescence microscopy using a mAb that reacts with NCTC 9343 PSB/4 (Fig. 4). The zwitterionic amino sugar-containing NCTC 9343 PSA/2, which is involved in abscess formation (Coyne *et al.*, 2001) and which also protects animals from experimental colitis (Mazmanian *et al.*, 2008), is not conserved amongst the three sequenced strains and is present in NCTC 9343 alone (Fig. 2a). Kuwahara *et al.* (2004) suggested that the presence of putative aminotransferase and dehydrogenase genes in five of the YCH46 PS loci could potentially generate five different abscess-inducing PSs. On this basis, four of the 638R loci that contain putative aminotransferases and dehydrogenases may produce a different set of abscess-inducing PSs.

### Within-strain phase-variable LC and SC comparisons

**Fig. 5.**
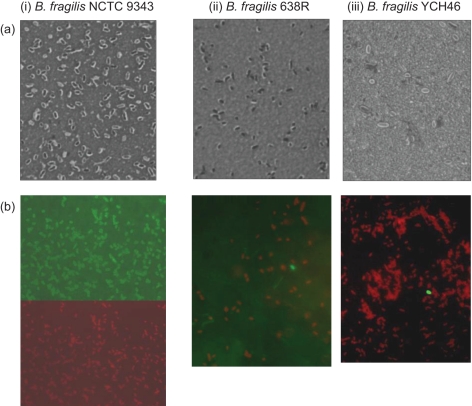
Light micrographs of eosin/carbol fuchsin negative stain (a) and immunofluorescence micrographs of bacteria reacted with anti-NCTC 9343 SC mAb followed by anti-mouse FITC-conjugated secondary antibody counter-stained with Evans blue (b). (i) *B. fragilis* NCTC 9343, (ii) 638R and (iii) YCH46 sampled from the 20–40 % interface of a Percoll step density gradient after centrifugation (×100 objective). (b, i) The same microscope field illustrating antibody labelling (top) and Evans blue counter stain (bottom); (b, ii) and (b, iii), combined fields. A small clear area can be observed surrounding the cells of *B. fragilis* NCTC 9343 and YCH46, indicating the expression of an SC. The capsule surrounding *B. fragilis* 638R appears more irregular. Note that 638R and YCH46 SC populations are not enriched for expression of the NCTC 9343 SC-associated epitope.

As with antigenic variation of the MC, phase variation of the LC and SC in NCTC 9343 occurs during growth of a single population in which both phase ON and phase OFF bacterial cells are evident. Neither 638R nor YCH46 produces an LC when cultured in a glucose DM (Van Tassell & Wilkins, 1978); nor is it possible to enrich for an LC population using Percoll density-gradient centrifugation (Patrick & Reid, 1983; Fig. 5). Three key genes are implicated in LC production by *B. fragilis* NCTC 9343: BF2782, a putative initiating glycosyltransferase WbaP homologue; BF2783, a putative Wza polysaccharide export/assembly protein homologue; and BF2784, a putative polysaccharide co-polymerase Wzc homologue.

**Fig. 6.**
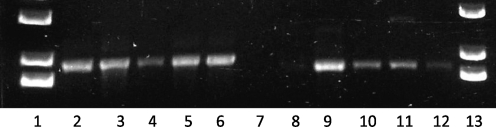
PCR products obtained from genomic DNA extracted from Percoll-purified MC, SC, LC *B. fragilis* NCTC 9343 populations, and mixed-capsule populations from *B. fragilis* 638R and YCH46, using primer pairs designed to amplify a 1.85 kb product comprising the complete BF2782 gene with its promoter in the ‘ON’ orientation (lanes 2–6) and a 1.75 kb product comprising the complete BF2782 gene with its promoter in the ‘OFF’ orientation (lanes 8–12). Lanes: 2 and 8, NCTC 9343 LC PCR; 3 and 9, NCTC 9343 SC PCR; 4 and 10, NCTC 9343 MC PCR; 5 and 11, 638R PCR; 6 and 12, YCH46 PCR; 1 and 13, 1 kb Plus DNA ladder (Invitrogen). Lane 7 is empty. Note the lack of product with the OFF orientation primers in the NCTC 9343 LC population, lane 8.

In NCTC 9343, expression of the LC is under the control of an invertible promoter, driven by Tsr19 (BF2780), a tyrosine site-specific recombinase (Chatzidaki-Livanis *et al.*, 2008). ACT comparisons indicated that the invertible promoter and the recombinase are conserved in 638R and YCH46. Comparison with the published YCH46 sequence, however, revealed that duplication of the inverted repeats present at one end of the NCTC 9343 invertible region is apparent at both ends of the invertible promoter region in YCH46. How this affects inversion of this region is not known. The invertible promoter region which mediates this within-strain variable expression of the LC is in the ‘OFF’ orientation in all three genome sequences. This is to be expected in the NCTC 9343 genome, as the DNA used for the sequencing project was purified from a population in which fewer than 1 % of the cells had the LC phenotype (Cerdeño-Tárraga *et al.*, 2005); therefore, it is unlikely that inversion would be evident in the shotgun sequencing. To determine whether active inversion occurred within the populations, PCR amplification of purified genomic DNA from populations of the three strains, using primer pairs designed to amplify the BF2782-equivalent gene with its promoter in both the ON and OFF orientations, resulted in the detection of amplicons of the predicted size for both orientations (Fig. 6). The promoter is therefore present in both orientations in populations of all three strains. This indicates the potential for phase-variable expression of the downstream genes, the first of which is the putative WbaP initiating glycosyltransferase. Wza proteins form major multimeric ring-like structures in the outer membrane that are thought to function as export channels in translocation of HMMPS across the outer membrane, and possibly also in assembly of the capsule on the cell surface (Drummelsmith & Whitfield, 2000); a BF2782 deletion mutant generated in NCTC 9343 does not exhibit the LC phenotype (Patrick *et al.*, 2009). The equivalent gene in 638R, BF638R 2790, contains a single nucleotide polymorphism (SNP) that results in a stop codon, located within the 5′ region of a putative C-terminal sugar transferase domain, potentially resulting in the truncation of approximately 75 % of this functionally important domain. This stop mutation may therefore abolish the enzymic function of the glycosyltransferase, which may in turn account for the lack of LC production by 638R.

Complementation of an NCTC 9343-BF2782 deletion mutant with pVA2198-BF2782, containing the invertible promoter in the ON position, restored the phase-variable LC phenotype (Patrick *et al.*, 2009); however, transformation of 638R with this plasmid did not result in an LC phenotype. It is possible that the stop codon in BF638R 2790 has polar effects on transcription of the additional downstream genes, although this will require confirmation by analysis of transcripts.

The reason for the lack of production of the LC in YCH46 is not immediately evident from the genome sequence, as the BF2782-equivalent gene (BF YCH46 2767) has an identical sequence to that of NCTC 9343. There are, however, SNPs in the downstream putative Wza BF YCH46 2768 (BF2783 equivalent), and in BF 638R 2793, equivalent to BF2784, the putative Wzc polysaccharide co-polymerase. These SNPs effect amino acid substitutions. Whether or not these alter the structure and biological function of the key capsular biosynthesis proteins remains to be determined. It may be that the lack of LC phenotype and presence of SNPs reflect the degree of laboratory subculture of these strains away from the selective pressures of the human host. All recent clinical and GI tract isolates of *B. fragilis* (*n*=32) examined to date produced the LC in proportions varying from 30 to 1 % or less (results not shown).

Populations of NCTC 9343 expressing an SC can be obtained at the 20–40 % interface layer of a Percoll discontinuous density gradient after centrifugation (Patrick *et al.*, 1986). Strains 638R and YCH46 also had an observable SC by light microscopy after negative staining, when sampled from the 20–40 % interface, although the SC was more irregular in strain 638R (Fig. 5). Labelling of the 638R and YCH46 SC populations with a mAb specific for the SC of NCTC 9343 (Reid *et al.*, 1987), however, revealed that these populations were not enriched for the NCTC 9343 SC-associated epitope (Fig. 5). As previously noted for NCTC 9343 (Patrick *et al.*, 1986), the SC population of strain 638R could be enriched by subculture from the 20–40 % Percoll interface layer into glucose DM; however, YCH46 failed to subculture. This may relate to the poor growth of YCH46 in glucose DM. YCH46 is growth-limited in this medium when compared with NCTC 9343 and 638R with respect to growth rate (mean doubling time of approximately 4 h as opposed to 1.6 and 1.2 h, respectively) and total viable count attained in late-exponential phase.

### Comparison of two PS loci yet to be associated with capsule type

**Fig. 7.**
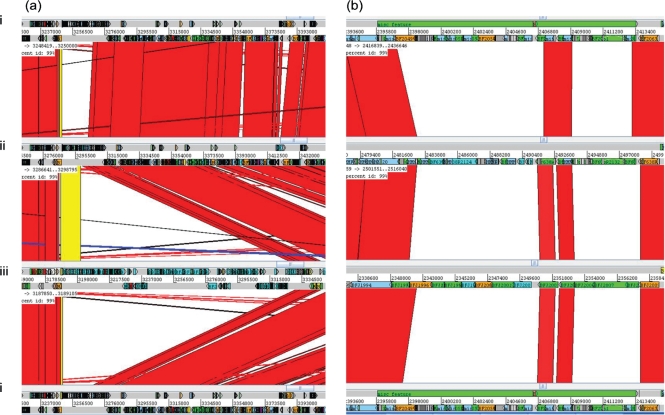
Four-way ACT genome comparison of PS-associated loci. (a) PSI/9: black arrows, inserted conjugative transposon-associated regions in YCH46; (b) PSK/11 of *B. fragilis.* (i) NCTC 9343, (ii) 638R, (iii) YCH46. Red or yellow coloration between sequences indicates sequence identity, no colour indicates divergent sequence.

The PS loci I/9 and K/11 are two further divergent loci that appear to lack invertible promoters and have yet to be associated with a particular capsular type (Fig. 7, Table 1).

In PS locus I/9 (Fig. 7a), comparison of 638R and YCH46 reveals conservation in a 12-gene region (from BF YCH46 2776 and BF 638R 2800) downstream of a predicted promoter, which is divergent in NCTC 9343. In YCH46, an inserted conjugative transposon disrupts the twelfth gene, BF YCH46 2787, a putative fucosyltransferase. This may inhibit or prevent synthesis of the PSI/9 PS or simply alter the structure. Interestingly, the next gene immediately downstream of BF YCH46 2787 in the locus is a putative recombinase. If the transposon is mobile, insertion and loss could generate variable expression of the putative PS encoded at this locus.

In addition, two acetyltransferase-related proteins within the PSI/9 region, otherwise conserved between YCH46 and 638R, are disrupted in 638R. A point mutation in BF638R 2810 generates a stop codon. In addition, in the BF638R region equivalent to BFYCH46 2780, a frameshift is generated by an additional cytosine in a run of 4 Cs. The presence or absence of an *O*-acetyl group on a sugar residue is a well-documented mediator of capsular variation in other bacteria. For example, *Escherichia coli* slipped-strand mispairing/replicative slippage can occur during DNA replication and results from heteropolymeric repeats in the region upstream of an *O*-acetyltransferase; this generates within-strain antigenic variation of the K1 PS (Vimr & Steenbergen, 2006). Homopolymeric repeat regions, within and outwith coding sequences, are also linked to variation generated by slipped-strand misparing/replicative slippage in other bacteria (van der Woude & Baumler, 2004). A mechanism such as this could generate antigenic variation of a PS encoded by this locus in strain YCH46.

In NCTC 9343, the PSI/9 locus, equivalent to the 12 genes of YCH46 and 638R, encodes an entirely different set of 18 genes associated with PS synthesis (from NCTC 9343 BF2791), indicating potential production of a different PS.

All three strains encode a putative Wzx flippase (divergent in NCTC 9343) in this region, and there are genes containing 8–12 transmembrane segments, characteristic of Wzy polymerases (Samuel & Reeves, 2003). This suggests that these PSs can be synthesized by an *E. coli* group 1 or 4 K antigen-like mechanism (Whitfield, 2006). The 10 genes in the region downstream are conserved amongst the three strains, and include a number of putative exported and membrane-associated protein genes. This region may be relevant to the production of sugar precursors for PS synthesis. Notably, there is a conserved *B. fragilis* TAnnTTTG consensus promoter (Bayley *et al.*, 2000) upstream of these 10 genes, indicating possible independent transcription.

PS K/11 is a novel locus, not previously reported, but present in all three sequenced strains. Again, there is considerable sequence divergence in this region (Fig. 7b). It lacks any upstream putative transcriptional regulator genes but contains a putative Wzy polymerase (BF2060 in NCTC 9343) and a putative Wzx flippase (BF2055 in NCTC 9343). This again suggests that these PSs could be synthesized by an *E. coli* group 1 or 4 K antigen-like mechanism (Whitfield, 2006).

It remains to be determined whether the PSI/9 and PSK/11 loci, which have yet to be assigned to a capsular structure, are related to expression of the LC or SC. The locus of fragilis glycosylation (*lfg*) region (Fletcher *et al.*, 2009), involved in protein glycosylation, is conserved amongst the three strains (Table 1). The potential influence of other conserved genes, putatively involved with PS export but not situated within PS biosynthesis-associated loci, such as BF2000 in NCTC 9343, is unknown.

### PS locus comparison amongst the Bacteroidetes

Xu *et al.* (2007) compared capsular PS loci in single genome sequences of *B. vulgatus*, *Bacteroides* *thetaiotaomicron* and *P. distasonis*, which are members of the Bacteroidetes associated with the human GI tract, but are infrequently isolated from infections. They identified nine PS-associated loci in *B. vulgatus*, eight in *B. thetaiotaomicron* and 13 in *P. distasonis*. In the current study, ACT comparisons confirmed the lack of conservation with the 10 *B. fragilis* PS loci (results not shown). Xu *et al.* (2007) reported that phage insertions disrupt four *P. distasonis* capsular PS loci, including the insertion of genes between the upstream regulatory genes (*upcY* and *upcZ*) and the downstream PS biosynthesis gene cluster of capsular PS locus 5 in *P. distasonis*. Five capsular PS biosynthesis loci of *B. vulgatus* are affected by the same phage, while two capsular loci are affected in *B. thetaiotaomicron* (Xu *et al.*, 2007). The presence of a conjugative transposon inserted within one of the *B. fragilis* PS loci, YCH46 PSI/9, is another mechanism that may mediate horizontal gene transfer (Fig. 7). Conjugative transposons have been reported to be associated with the duplication and translocation of *B. vulgatus* PS loci (Xu *et al.*, 2007).

### DNA restriction and modification (R-M) systems

The difficulties and low efficiency of successful genetic manipulation of *Bacteroides*, based on the introduction of foreign DNA via electroporation, are well documented (e.g. Smith, 1995). R-M systems are proposed to protect a bacterial cell against incoming foreign DNA, particularly invading bacteriophages, but could have similar effects on DNA introduced for the purpose of genetic manipulation. The human GI tract contains a significant number and diversity of phages, as shown by metagenomic studies (Breitbart *et al.*, 2003). Bacteriophages may have been a significant driving force in relation to the evolution of the diversity of surface phenotypes described above. The genome of *B. fragilis* NCTC 9343 specifies three type I and two type III R-M systems, as compared with the 638R genome, which specifies two type I, one type IIS and one type III R-M systems.

Type I R-M systems consist of three subunits, HsdR, HsdM and HsdS, the last of which dictates the DNA-binding specificity of the methyltransferase and the endonuclease. The NCTC 9343 genome contains a locus (BF1839–1842) which shows phase-variable expression of an HsdS subunit with eight different possible polypeptide combinations, which in turn provides eight different DNA-recognition specificities (Shufflon BB). A related *hsdS* shufflon (BF 638R 1146–1149) is present in the genome of 638R, but is located in a different chromosomal location, and the encoded polypeptides share between 27 and 49 % identity with the products of the NCTC 9343 shufflon. This indicates that the shufflons have a common origin, although the coding sequences have subsequently diverged.

In NCTC 9343, the shufflon contains four sets of inverted repeats that are associated with site-specific rearrangement of the target recognition domains (TRDs) within the encoded HsdS proteins. One of these repeats (5-′CTTATTGATGAACGTATCGCTACCCAAAACAAAATCAT-3′) is conserved (32/38 bp) within the 638R shufflon, suggesting that a common recombinase is responsible for inversion for at least two of the TRDs. In the shufflons of both genomes, genes encoding tyrosine integrases with 61 % identity are present immediately adjacent to the last *hsdS* gene, reinforcing the notion that they mediate this subset of inversions. Both type I R-M systems in 638R are contained within a putative 50 kb horizontally acquired island, ICE2, which is possibly a prophage relic. The type IIS R-M bifunctional enzyme is encoded within a chromosomal island in the analogous position to the island that encodes the type I shufflon in NCTC 9343. The type III-specifying locus in 638R (BF 638R 1130 and 1131) is the only common R-M system amongst the three sequenced strains of *B. fragilis*. Together, these observations suggest that the common genome backbone of *B. fragilis* specifies a type III R-M system and that diversification of R-M specificities within strains is achieved by horizontal gene transfer.

The extent and variety of R-M systems in 638R will act to prevent phage infection but will also be a barrier to horizontal gene transfer. At least two of the conjugative transposons that reside in the genome have evaded this barrier by encoding homologues of the ArdA anti-restriction protein that mimics B-form DNA and blocks the binding site of type I R-M systems (McMahon *et al.*, 2009).

In addition to the classical R-M systems, the NCTC 9343 genome encodes proteins with homology to the type IV restriction endonuclease McrBC, which cleaves specific DNA sequences modified with 5-methylcytosine. These genes are not present in the genome of 638R, and their absence, despite the potential expression of nine different type I R-M specificities, might contribute to the successful transformation of the strain using DNA derived from *E. coli* K-12 that is usually modified by the Dcm methyltransferase.

The R-M systems encoded within *B. fragilis* are undoubtedly responsible for the low efficiency of successful genetic manipulation based on the introduction of foreign DNA via electroporation, in particular in relation to NCTC 9343 (e.g. Smith, 1995). This is also in keeping with the observed improvement of electroporation efficiency when the bacteriophage T7 Ocr (overcomes classical restriction) protein, a DNA mimic, is included in the electroporation mix (Patrick *et al.*, 2009). Ocr protein reduces the activity of type I R-M systems in a range of bacteria and archaea (Dryden, 2006).

### General genomic comparisons

The general characteristics of the three genomes are broadly similar (Table 2). Regions of low or no sequence identity amongst the three individual genomes account for approximately 11 % of the genome sequences. These non-identical regions include the PS regions as discussed above, and differences in inserted extra-chromosomal elements (ICEs) such as conjugative transposons and prophages. Differences in numbers of ICEs also largely account for differences in chromosomal size between the strains.

#### Invertible regions.

**Fig. 8.**
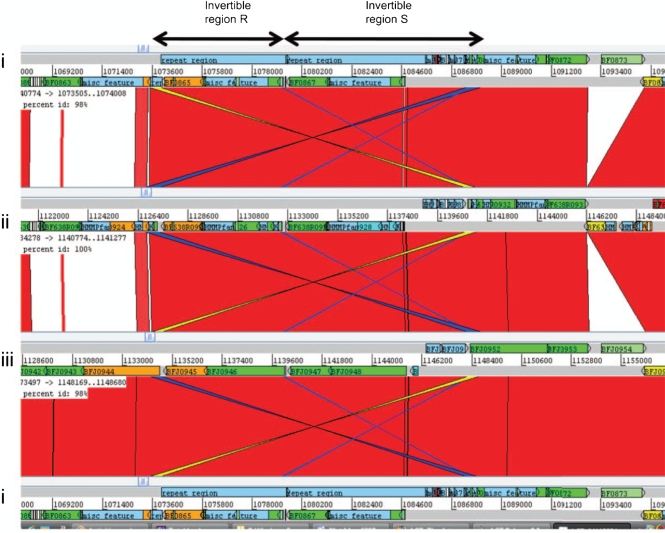
Comparison of invertible regions R and S (arrows), active in the NCTC 9343 shotgun sequence of *B. fragilis.* (i) NCTC 9343, (ii) 638R, (iii) YCH46. Red coloration between sequences indicates sequence identity, no colour indicates divergent sequence, dark blue/yellow indicates inverted sequences. Note the sequence identity of inverted sequences in R and S, which suggests that these two regions form a complex intergenic shufflon.

A major unusual feature of the *B. fragilis* genome, first identified in NCTC 9343, is the diversity and extent of invertible DNA elements within the genome. These include 16 fragilis invertible (fin) promoter regions, in addition to those controlling PS loci and three complex invertible DNA shufflons (Cerdeño-Tárraga *et al.*, 2005). Conserved fin regions, not associated with PS loci, are evident throughout all three genomes. Invertible regions R and S, which involve putative outer-membrane proteins, are present in all three strains. An overlap of inverted repeat regions in R and S is evident in the comparison of the three genomes, suggesting that these two regions together may generate multiple rearrangements, thus forming a further complex gene shufflon (Fig. 8). The invertible region Q, which causes alteration of the orientation of two hypothetical proteins with or against the direction of transcription of the surrounding genes, is, however, missing from 638R, as is the complete putative phage-associated region. The previously described complex invertible region shufflons CC and EE in NCTC 9343 (Cerdeño-Tárraga *et al.*, 2005) are present in both 638R and YCH46, although only in NCTC 9343 does the shufflon EE contain an inserted conjugative transposon. The function of these many potentially variable surface proteins is unknown, but it would be interesting to determine whether they are linked to bacteriophage adsorption.

#### Origin of 638R rifampicin resistance.

The spontaneous rifampicin-resistant mutant strain 638R (Privitera *et al.*, 1979) was generated at the Institut Pasteur from a clinical isolate originating from Chicago (Stiffler *et al.*, 1974). Rifampicin binds within the DNA/RNA channel of the *β* subunit in RNA polymerase and blocks the exit path for the nascent transcript. There is a single putative *rpoC* RNA polymerase *β* subunit homologue in all three *B. fragilis* genomes. Comparison of the nucleotide sequence reveals 12 single base pair changes; however, only one results in an altered amino acid. A transition at base pair 1475 results in the loss of a conserved serine, which is replaced with a phenylalanine. Serine 492 in Bf-RpoC corresponds to serine 411 in the *Thermus aquaticus* (*Taq*) RNA polymerase, which forms a hydrogen bond with oxygen 2 in the naphthol ring of rifampicin (Campbell *et al.*, 2001). Mutations that change serine 411 to phenylalanine in *Taq* RNA polymerase make the enzyme insensitive to rifampicin; this is also true for the corresponding S531F change in the *β* subunit of *E. coli* RNA polymerase (Campbell *et al.*, 2005).

### Conclusion

Each *B. fragilis* strain examined has 10 separate divergent PS regions, each potentially capable of generating an extracellular PS, of which only two are conserved between two of the strains. There is, therefore, the potential for the expression of 28 different PSs amongst these three strains. This represents an unprecedented diversity of PSs within such a limited number of strains. For example, in *Streptococcus pneumoniae*, although there are 90 different PS loci, each confers a single serotype on a single strain (Bentley *et al.*, 2006).

Surface component variation may enhance survival when *B. fragilis* escapes into other body sites and generates opportunistic infection, but this does not fully explain why *B. fragilis* is more commonly associated with opportunistic infections, including bacteraemia, when compared with other members of the Bacteroidetes. Undoubtedly a number of factors in combination will contribute to virulence. For example, a fibrinogen-binding protein identified in NCTC 9343 (Houston *et al.*, 2010) is conserved amongst the three sequenced *B. fragilis* strains NCTC 9343, 638R and YCH46, which were isolated from abscess and bacteraemia patients, but is not conserved in *B. vulgatus* and shares only 33 % identity to a putative cell surface antigen of *B. thetaiotaomicron* (BT_1896), both of which are rarely isolated from clinical infections.

The multiple and diverse PS biosynthesis loci in *B. fragilis* and other members of the Bacteroidetes indicate a strong selective pressure in favour of PS variation within the human GI tract. It is likely that these have arisen from a divergence of operons over evolutionary time combined with more rapid changes arising from horizontal transmission amongst cells representing a pan-genome with an extensive pool of different PS loci. Interestingly, horizontal gene transfer has occurred despite the presence of a significant barrier in the form of diverse and phase-variable DNA R-M systems.

Given the number of phage-related regions within the *B. fragilis* genome, and the presence of diverse PS loci in Bacteroidetes infrequently associated with opportunistic infection, such as *B. vulgatus* and *P. distasonis*, it may be that a major driver of amongst-strain PS locus diversity, within-strain surface protein and PS diversity generated by DNA inversion (Cerdeño-Tárraga *et al.*, 2005), and extensive DNA restriction and modification, is the prevention of bacteriophage adsorption and infection within the GI tract.

## Figures and Tables

**Table 1.** PS biosynthesis-associated loci in *B. fragilis* NCTC 9343, 638R and YCH46

**Locus**	CDSs*			**Phenotype association**
	**NCTC 9343**	**638R**	**YCH46**	
A/2	1369−1377	1435–1460	1430–1445	MC
B/4	1895−1914	1864–1883	1830–1848	MC
C/8	1011−1026	1076–1095	1094–1113	MC
D/5	3682−3697	3750–3773	3911–3923	MC
E/1	2591−2604	2585–2598	2566–2583	MC
F/6	1551−1565	1539–1563	1530–1551	MC
G/7	0733−0752	0778–0810	0805–0827	MC
H/3	3451−3464	3472–3487	3646–3665	MC
I/9	2791−2817	2800–3826	2776–2935	Unknown
J/10	1706−1710	1710–1714	1700–1704	MC
K/11	2048−2062	2121–2133	1995–2008	Unknown
LC assembly	2782−2784	2790–2793	2767–2769	LC
*lfg*†	4298−4306	4457–4465	4504–4512	Protein glycosylation

*Coding sequences. †Locus of fragilis glycosylation.

**Table 2.** General genomic comparison of *B. fragilis* NCTC 9343, 638R and YCH46

**Parameter**	**Strain**		
	NCTC 9343*	**638R**	**YCH46†**
Genome size (bp)	5 205 140	5 373 121	5 277 274
CDSs‡	4241	4308	4578
Plasmid	pBF9343 (36.6 kb)	0	pBFY46 (33.7 kb)

*Cerdeño-Tárraga *et al.* (2005). †Kuwahara *et al.* (2004). ‡Coding sequences.

## Abbreviations

- ACT, Artemis Comparison Tool
- GI, gastrointestinal
- HMMPS, high-molecular-mass polysaccharides
- LC, large capsule
- MC, micro-capsule
- PS, polysaccharide
- R-M systems, restriction and modification systems
- SC, small capsule
- SNP, single nucleotide polymorphism
